# Cancer-associated fibroblasts promote the progression of endometrial cancer via the SDF-1/CXCR4 axis

**DOI:** 10.1186/s13045-015-0231-4

**Published:** 2016-02-06

**Authors:** Fei Teng, Wen-Yan Tian, Ying-Mei Wang, Yan-Fang Zhang, Fei Guo, Jing Zhao, Chao Gao, Feng-Xia Xue

**Affiliations:** Department of Gynecology and Obstetrics, Tianjin Medical University General Hospital, NO 154, Anshan Road, He Ping District, Tianjin, 300052 China

**Keywords:** Tumor microenvironment, Cancer-associated fibroblasts, Endometrial cancer, Stromal cell-derived factor-1α, CXCR4, Prognosis

## Abstract

**Background:**

Cancer-associated fibroblasts (CAFs) are believed to play an essential role in cancer initiation and development. However, little research has been undertaken to evaluate the role of CAFs in endometrial cancer (EC) progression. We aim to detect the functional contributions of CAFs to promote progression of EC.

**Methods:**

Stromal fibroblasts were isolated from endometrioid adenocarcinomas and normal endometrial tissues. The conditioned media of cultured CAFs and normal fibroblasts (NFs) were collected to detect the level of stromal cell-derived factor-1alpha (SDF-1α), macrophage chemoattractant protein-1 (MCP-1), migration inhibitory factor (MIF), colony stimulating factor-1 (CSF-1), and interleukin-1 (IL-1) by ELISA. The CAFs or NFs were cocultured with EC cell lines to determine the proliferation, migration, and invasion by MTT assays and transwell chambers. Xenograft models were used to observe tumor growth. Matrix metalloproteinases (MMP)-2 and MMP-9 activity was evaluated by zymography. AMD3100 (a chemokine receptor 4 (CXCR4) antagonist) was used to block the SDF-1/CXCR4 axis. Neutralizing antibodies were used to detect PI3K/Akt and MAPK/Erk pathways by western blotting. SDF-1α and CXCR4 expressions were analyzed in xenotransplanted tumors and 348 cases by immunohistochemistry.

**Results:**

CAFs promoted proliferation, migration, and invasion as well as in vivo tumorigenesis of admixed EC cells significantly more than NFs by secreting SDF-1α. These effects were significantly inhibited by AMD3100. CAFs promoted EC progression via the SDF-1α/CXCR4 axis to activate the PI3K/Akt and MAPK/Erk signalings in a paracrine-dependent manner or increase MMP-2 and MMP-9 secretion in an autocrine-dependent manner. SDF-1α and CXCR4 expression upregulation accompanied clinical EC development and progression. High SDF-1α expression levels were associated with deep myometrial invasion, lymph node metastasis, and poor prognosis in EC.

**Conclusions:**

Our data indicated that CAFs derived from EC tissues promoted EC progression via the SDF-1/CXCR4 axis in a paracrine- or autocrine-dependent manner. SDF-1α is a novel independent poor prognostic factor for EC patients’ survival. Targeting the SDF-1/CXCR4 axis might provide a novel therapeutic strategy for EC treatment.

## Background

Endometrial cancer (EC) is the most common gynecological malignancy in developed countries, with approximately 52,630 new cases and 8590 deaths occurring in the USA in 2014 [1]. With changes in Western lifestyles and the rising prevalence of obesity in developing countries, the incidence of EC has increased at a surprisingly rapid rate in China [2]. For patients with advanced or recurrent disease or for those who wish to preserve their fertility, the treatment options are limited. Therefore, it is of great urgency to understand the mechanisms of EC so that specific therapeutic targets can be designed and developed.

Solid tumors, including EC, consist of tumor cells and various types of stromal cells; thus, tumor progression is determined not only by the tumor cells themselves but also by the tumor stroma. Previous studies have shown that the crosstalk between tumor cells and their surrounding stroma plays an important role in tumor development [3, 4]. Myofibroblasts in the tumor stroma, collectively called cancer-associated fibroblasts (CAFs), are large, spindle-shaped mesenchymal cells that share characteristics with smooth muscle cells that expressed both vimentin and alpha-smooth muscle actin (α-SMA) [5]. CAFs also exhibit similar phenotypes with mesenchymal stem cells; both cell types secrete similar cytokines [6], while mesenchymal stem cells could produce large amounts of exosomes [7]. The presence of CAFs was proposed to precede tumor cell initiation, proliferation, invasion metastasis, angiogenesis, and chemoresistance in vitro experiments in prostate cancer, pancreatic cancer, gastric cancer, and breast cancer [8–12]. However, the mechanisms linking CAFs and cancer progression are not fully understood.

CAFs can secrete variety of factors, such as cytokines, chemokines, and growth factors. These soluble factors are involved in paracrine signaling or in autocrine loops to contribute to tumor progression [13], stromal cell-derived factor-1alpha (SDF-1α) (CXCL12) is a member of the CXC chemokine family, the chemotactic effects of which are mediated by interaction with chemokine receptor 4 (CXCR4). Typically, SDF-1, which is expressed in the stromal fibroblasts of some organs, including the brain, breasts, liver, lungs, and lymph nodes, is involved in carcinoma survival, proliferation, and metastasis [14–16]. Malgorzata et al. found that increased expression of SDF-1 was correlated with a more aggressive phenotype and a negative prognostic factor of EC [17]. In addition, previous studies showed that CAFs secrete SDF-1, activate SDF-1/CXCR4 axis, and promote tumor progression [18, 19]. Other researchers have also showed that blocking the interaction between SDF-1 and CXCR4 could inhibit the number or size of tumor metastases [20–23]. Therefore, we investigated whether CAFs played a role in promoting the progression of EC by secreting SDF-1.

To test this hypothesis, we established several primary cultures of CAFs and normal fibroblasts (NFs) from EC tissues and normal endometrial tissues, and then, we cocultured CAFs or NFs with EC cell lines in vivo and in vitro. We concluded that, in contrast to NFs, CAFs promoted EC cell proliferation, migration, and invasion. We measured the levels of SDF-1α in the conditioned media harvested from NFs and CAFs. We found that CAF-mediated EC progression was modulated via the SDF-1/CXCR4 axis, which activated intracellular PI3K/Akt and/or MAPK/Erk signalings and increased active matrix metalloproteinases (MMP)-2 and MMP-9 expressions. Furthermore, we determined SDF-1α and CXCR4 expression levels and their relationships with clinicopathologic features and clinical outcomes in human EC patients. This study provides new evidence elucidating the pro-tumorigenic role of CAFs in the progression of EC.

## Results

### Establishment of primary fibroblast cells

**Fig. 1:**
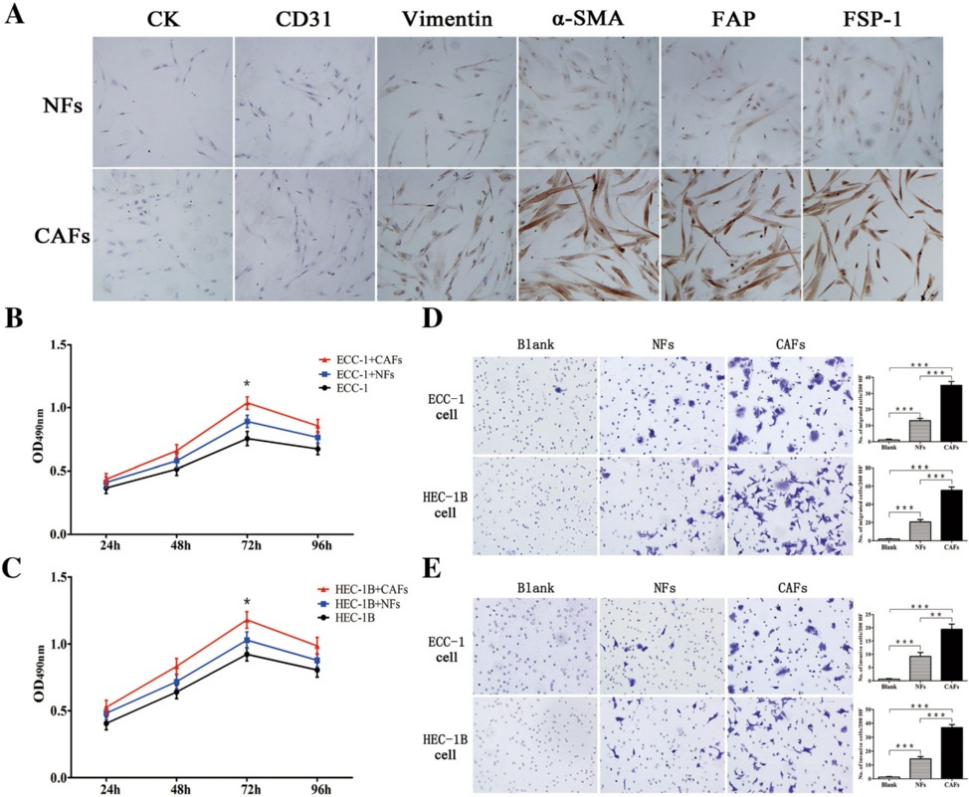
Identification of stromal fibroblasts and CAFs promoting EC cell proliferation, migration, and invasion. **a** Identification of primary cultured fibroblast cells by immunocytochemistry (magnification ×400). **b**, **c** Cell proliferation was measured by MTT assay. **d**, **e** Crystal violet staining for migrated and invaded EC cells (magnification ×200). Three independent experiments were performed in triplicate. Data are expressed as means ± SDs. *P < 0.05; **P < 0.01; ***P < 0.001

To establish primary fibroblast cells, normal endometrial tissues or EC tissues were digested with collagenase and hyaluronidase. The primary fibroblast cells displayed elongated spindle-shaped features. The fibroblast cells were verified using anti-vimentin, anti-α-SMA, anti-fibroblast-activating protein (FAP), and anti-fibroblast-specific protein (FSP)-1 antibodies. The antibody to CD31 was used to demonstrate a lack of endothelial cell contamination, while cytokeratin (CK) was used to exclude epithelial components. CAFs isolated from EC tissues were negative for CK and CD31 expression, moderately positive for vimentin expression, but highly positive for α-SMA, FSP-1, and FAP expression. NFs isolated from normal endometrial tissues showed negative expression of CK and CD31 and moderate expression of vimentin, α-SMA, FSP-1, and FAP. These findings are indicating that the isolated fibroblast cells were relatively pure and free of epithelial cells or endothelial cells contamination (Fig. 1a).

### CAFs promoted EC cell growth and tumorigenesis

To investigate whether CAFs play an important role in the progression of EC, we conducted MTT assays to evaluate the proliferation of EC cell lines cocultured with CAFs or NFs for 4 days. Both CAFs and NFs promoted the proliferation of HEC-1B and ECC-1 cells in a time-dependent manner. CAFs, compared with NFs, had more prominent potential to promote HEC-1B and ECC-1 cell proliferation at 72 h (*P* < 0.05; Fig. 1b, c).

**Fig. 2:**
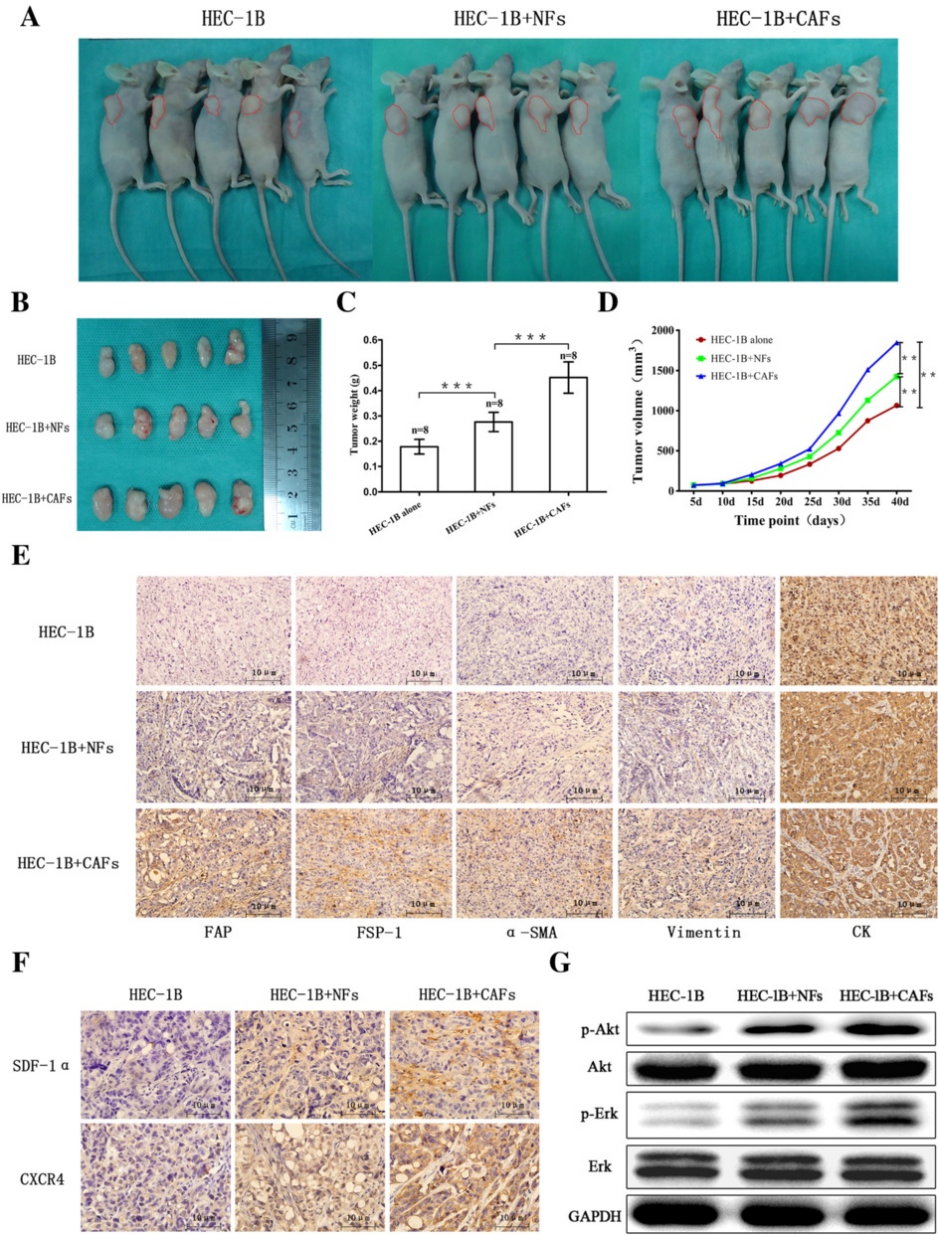
CAFs comingled with HEC-1B cells, enhancing xenograft growth in nude mice. **a**, **b** Gross appearance of xenografts; HEC-1B cells were injected alone or were coinjected with various fibroblasts subcutaneously into the right flanks of 4-week-old nude mice, and xenografts were harvested after 40 days of growth in female hosts. **c** Average tumor weight in each group was evaluated at 40 days after injection. *Error bars* depict the standard error around the mean. ***P < 0.001. **d** Tumor volume was plotted on the indicated days. **P < 0.01; ***P < 0.001. **e** Sections of xenografts in different groups were submitted to immunohistochemistry using antibodies specific for human CK, vimentin, α-SMA, FAP, and FSP-1. *Scale bar*, 10 μm. **f** Expression of SDF-1α and CXCR4 in xenotransplanted tumors. **g** Western blot analysis detection of the phosphorylation of Ark and Erk protein expression in xenotransplanted tumors

Next, to evaluate the contribution of CAFs to tumor growth in vivo, we developed a human tumor xenograft model that has both stromal and epithelial compartments in the engrafted tumors. We mixed CAFs or NFs with HEC-1B cells at a 3:1 ratio and inoculated these mixtures subcutaneously into the right flanks of immunodeficient nude mice. The tumor formation was monitored. HEC-1B cells co-mixed with CAFs generated tumors of greater volume and weight than HEC-1B cells mixed with NFs or HEC-1B cells alone (Fig. 2a–d). The stromal fibroblasts’ phenotypes were determined by immunohistochemistry using antibodies specific for human CK, vimentin, α-SMA, FAP, and FSP-1. The CAFs were negative for CK expression and weakly positive for vimentin and α-SMA expression but moderately positive for FSP-1 and FAP expression. The NFs showed negative expression of CK and weak expression of vimentin, α-SMA, FSP-1, and FAP (Fig. 2e). These results suggested that the human originated CAFs promoted the growth and tumorigenesis of EC.

### CAFs promoted EC cells migration and invasion

A coculture method was used to determine whether CAFs secreted factors that could stimulate the migration and invasion of EC cells in vitro. Our findings showed that the number of migrating ECC-1 cells increased by coculturing with CAFs, compared to coculturing with NFs or control medium (*P* < 0.001; Fig. 1d). Invasion followed the same pattern with migration; the number of invading cells was significantly greater for coculturing with CAFs, compared to coculturing with NFs or control medium (*P* < 0.001; Fig. 1e). Consistent results were obtained with HEC-1B cells (Fig. 1d, e).

### Cytokines were secreted by NFs/CAFs in the culture supernatant

**Fig. 3:**
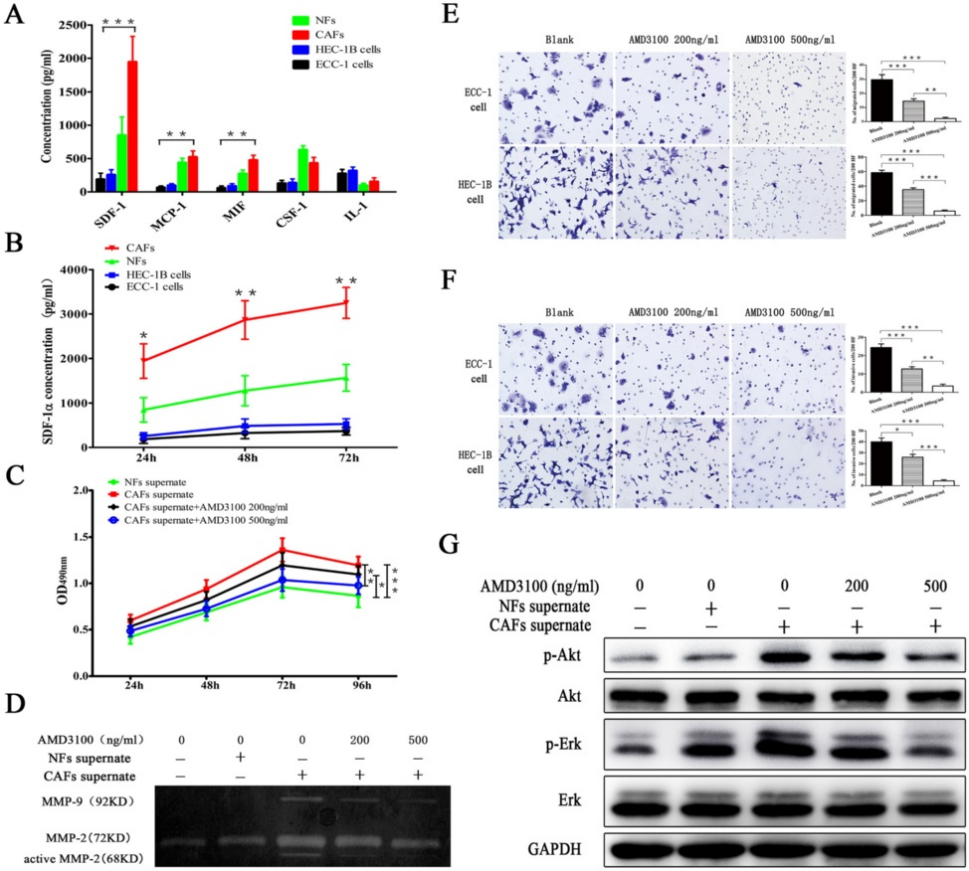
CAFs promoted EC progression via the SDF-1α/CXCR4 axis. **a** Cytokines secreted by EC cells, NFs, and CAFs were measured by ELISA. **b** SDF-1α protein concentration in the conditioned media was measured for 96 h by ELISA. **c** The effects of SDF-1α-induced proliferation of HEC-1B cells were inhibited by AMD3100. Three independent experiments were performed in triplicate. Data are expressed as means ± SDs. *P < 0.05; **P < 0.01; ***P < 0.001. **d** MMP-2 and MMP-9 gelatinase activity in the conditioned media was analyzed by zymography. **e**, **f** The effects of SDF-1α-induced migration and invasion of EC cells were inhibited by AMD3100 (magnification ×200). **g** Western blot analysis detection of the phosphorylation of Ark and Erk protein expressions in HEC-1B cells

To determine the factors responsible for CAF-mediated cell proliferation, migration, and invasion, we measured the levels of SDF-1α, macrophage chemoattractant protein-1 (MCP-1), migration inhibitory factor (MIF), colony stimulating factor-1 (CSF-1), and interleukin-1 (IL-1) in the conditioned media harvested from ECC-1 cells, HEC-1B cells, NFs, and CAFs. CAFs secreted greater amount of SDF-1α, MCP-1, and MIF when compared to NFs and EC cells. Of this class of cytokines, a significant higher level of SDF-1α than MCP-1 (approximately threefold) and MIF (approximately threefold) levels were secreted by CAFs (Fig. 3a). Therefore, we focused on the SDF-1α and tested the protein concentration in the conditioned media of EC cells, NFs, and CAFs for 24, 48, 72, and 96 h. This assay indicated that SDF-1α in the conditioned media from CAFs was significantly higher than that from NFs or EC cell lines (Fig. 3b).

### A crucial role for the SDF-1/CXCR4 axis in the promotion of EC progression

Finally, to determine whether SDF-1α secreted by CAFs was closely involved in the proliferation, migration, and invasion of EC cells, we treated EC cells with AMD3100 (a CXCR4 antagonist) in presence of CAF-conditioned media in vitro. SDF-1α-induced cell proliferation, migration, and invasion were significantly inhibited by AMD3100 pretreatment (Fig. 3c, e, f).

### MMP-2 and MMP-9 activity was regulated by the SDF-1/CXCR4 axis in an autocrine-dependent manner in CAFs

CAFs could dramatically increase the mobility of EC cells and their invasion through Matrigel. These effects of CAFs did not require direct cell-cell contact because the conditioned media of CAFs produced essentially the same effects. Correlated with this finding, conditioned media from HEC-1B cells, NFs, and CAFs were analyzed by zymography. Compared with conditioned media from with NFs or HEC-1B cells, conditioned media from CAFs resulted in increased MMP-2 and MMP-9 gelatinase activity. When CAFs were treated with AMD3100, MMP-2 and MMP-9 expression was reduced (Fig. 3d). These findings indicated that CAFs mediated the invasion and metastasis of EC via the SDF-1/CXCR4 axis in an autocrine-dependent mode.

### Activation of the PI3K/Akt and MAPK/Erk signalings in CAF-mediated EC cells proliferation

To elucidate the mechanisms underlying the growth-promoting effects of CAF secretion on EC, we determined the activation of PI3K/Akt and MAPK/Erk, two major signalings implicated in EC. HEC-1B cells were starved in serum-free medium for 12 h and incubated with DMEM/F12, the condition media of NFs and CAFs and CXCR4 antagonists (AMD3100 200 ng/ml, 500 ng/ml). HEC-1B cells were pretreated with AMD3100 for 1 h and incubated with the condition media of CAFs for 30 min. Phospho-Akt and phospho-Erk 1/2 protein levels were elevated when HEC-1B cells were treated with CAFs supernatant compared with NFs supernatant. Next, CXCR4 was blocked by its antagonist, and the phosphorylation of Akt and Erk 1/2 in response to SDF-1α was significantly decreased (Fig. 3g). In addition, we observed that the elevated phosphorylation of Akt and Erk 1/2 protein levels in the xenografts tumors containing CAFs compared to tumors without fibroblasts or containing NFs (Fig. 2g). These data suggested that the activation status of the PI3K/Akt and/or MAPK/Erk signalings might be the key point in CAF-mediated EC cells proliferation.

### Expression of SDF-1α in EC

**Fig. 4:**
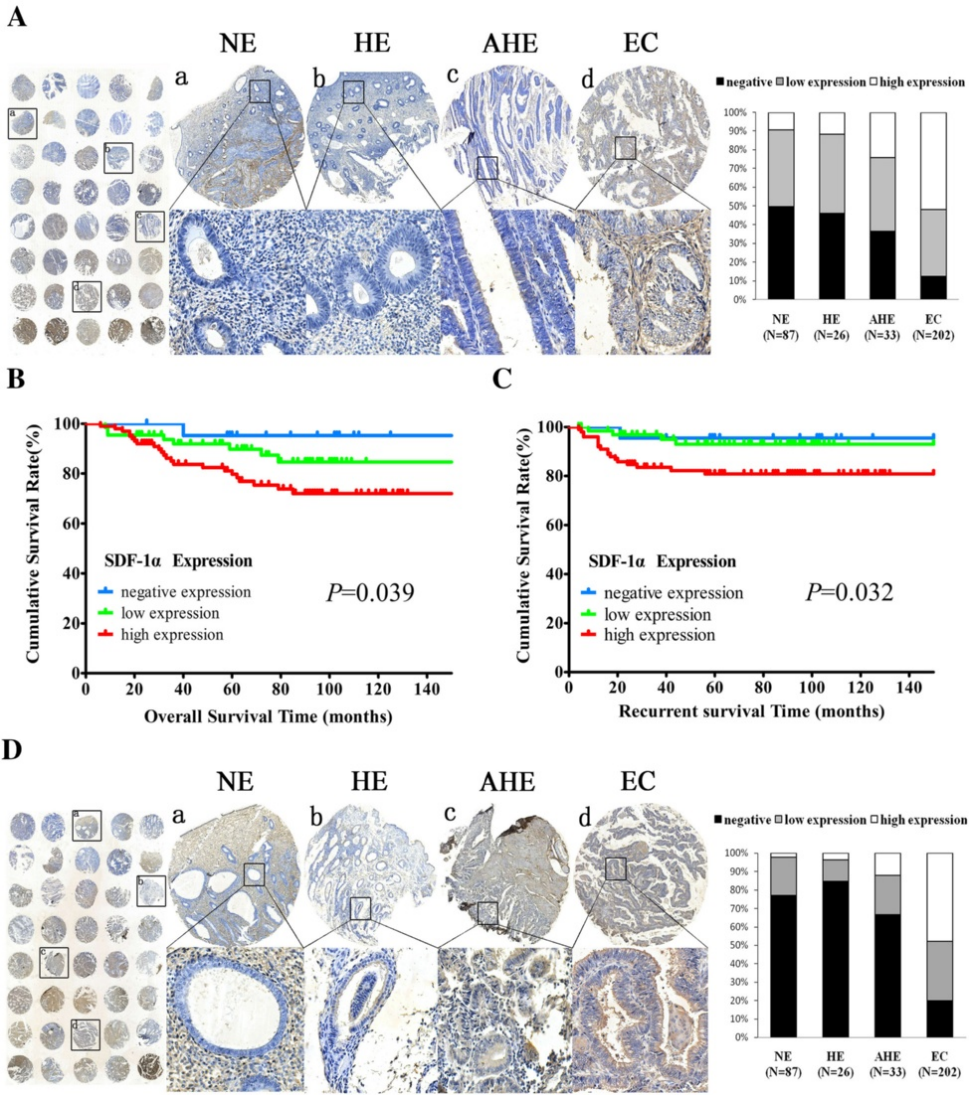
Expression of SDF-1α and CXCR4 in EC. **a** SDF-1α protein expression upregulation accompanied clinical EC development and progression. **b** Kaplan-Meier survival curves for overall survival according to SDF-1α staining level: negative expression, low expression, and high expression. Overall survival was significantly different in the three groups (*P* = 0.039). **c** Kaplan-Meier survival curves for recurrence according to SDF-1α staining level: negative expression, low expression, and high expression. Recurrent survival was significantly different in the three groups (*P* = 0.032). **d** CXCR4 expression increased gradually in endometrial tissues as they progressed from NE to HE to AHE and finally to EC tissues

To determine SDF-1α’s role in EC tumorigenesis and progression, we performed immunohistochemical analysis to evaluate SDF-1α expression in xenotransplanted tumors, as well as in normal endometrium (NE), hyperplastic endometrium (HE), atypical hyperplastic endometrium (AHE), and EC tissue microarrays. SDF-1α staining intensity was greater in the stromal compartment than in the endometrial epithelial compartment, and its staining was located in the membrane and/or cytoplasm. SDF-1α expression was stronger in the xenografts tumor containing CAFs compared to tumors without fibroblasts or containing NFs (Fig. 2f). Among the 348 cases available for analysis, SDF-1α expression was lowest in NE tissues; the levels were higher in HE and AHE tissues and highest in EC tissues (*χ*^2^ = 86.826, *P* = 0.000; Fig. 4a). More specifically, SDF-1α expression was significantly higher in EC tissues than in NE tissues (*P* = 0.000), in HE tissues (*P* = 0.000), and in AHE tissues (*P* = 0.000). There were significant differences in tissue SDF-1α expression between AHE and HE tissues (*P* = 0.000), whereas there were no significant differences in tissue SDF-1α expression between AHE and NE tissues (*P* = 0.233) or between HE and NE tissues (*P* = 0.923).

### Correlations between SDF-1α expression and clinicopathologic factors

We next analyzed the correlations between SDF-1α expression and various clinicopathologic factors that could affect the prognosis of patients with EC. Statistical analysis revealed that SDF-1α expression was significantly increased during the NE-HE-AHE-EC sequence in the 348 samples. The analyses revealed that, among the 202 EC patients, a high level of SDF-1α expression was associated with lymph node metastasis (*P* = 0.038) and deep myometrial invasion (myometrial invasion ≥1/2; *P* = 0.018). In contrast, SDF-1α expression was not correlated with age at diagnosis, menopausal status, histologic subtype, histopathologic grade, or advanced tumor stage (Table 1).

**Table 1:** Associations between tissue SDF-1α expression and clinicopathologic characteristics of EC patients

Variables	SDF-1α expression (*n* (%))			Total *N*	*χ* ^2^	*P*
	Negative	Low	High			
Age at diagnosis (years)						
<60	14 (11.2)	43 (34.4)	68 (54.4)	125	1.133	0.567
≥60	12 (15.6)	28 (36.4)	37 (48.0)	77		
Menopausal status						
Yes	15 (10.6)	53 (37.6)	73 (51.8)	141	2.604	0.272
No	11 (18.0)	18 (29.5)	32 (52.5)	61		
Histologic subtype						
Endometrioid	22 (11.9)	66 (35.7)	97 (52.4)	185	1.616	0.446
Nonendometrioid	4 (23.5)	5 (29.4)	8 (47.1)	17		
Histopathologic grade						
G1	14 (14.4)	34 (35.1)	49 (50.5)	97	1.460	0.834
G2	7 (9.2)	29 (38.2)	40 (52.6)	76		
G3	4 (13.8)	9 (31.0)	16 (55.2)	29		
Myometrial invasion						
<1/2	16 (10.9)	60 (40.8)	71 (48.3)	147	7.998	0.018^*^
≥1/2	10 (18.2)	11 (20.0)	34 (61.8)	55		
FIGO stage						
I–II	22 (13.2)	63 (37.7)	82 (49.1)	167	3.456	0.172
III–IV	4 (11.4)	8 (22.9)	23 (65.7)	35		
Lymph node metastasis						
Yes	1 (6.3)	2 (12.5)	13 (81.2)	16	6.530	0.038^*^
No	24 (12.9)	70 (37.6)	92 (49.5)	186		

The data are presented as numbers of cases (percentages of cases)

* indicates *P* < 0.05

### Correlation between SDF-1α expression and survival

To investigate further the clinical relevance of SDF-1α expression in EC, we compared overall survival according to SDF-1α expression using Kaplan-Meier estimates. A total of 15 patients were lost to follow-up; thus, the data from 187 patients were included in the analysis. Of the 32 (17.11 %) patients who died, 21 (65.62 %) died of cancer and 11 (34.38 %) died of other causes, namely heart failure, cerebral infarction, chronic diseases, and cerebral hemorrhage. Remarkably, regarding the 10-year survival rates in the negative SDF-1α expression group, 21 (95.45 %) patients survived, and 1 patient (4.55 %) died. In the low SDF-1α expression group, 57 (87.69 %) patients survived, and 8 patients (12.31 %) died. Finally, in the high SDF-1α expression group, 77 (77.00 %) patients survived, and 23 patients (23.00 %) died. These data suggested a better prognosis for the negative SDF-1α expression group than for the low expression and high expression groups (*χ*^2^ = 6.473, *P* = 0.039; Fig. 4b). Moreover, we also compared the correlation between SDF-1α expression and recurrent survival. Recurrence occurred in 23 of the 187 EC patients. In the negative SDF-1α expression group, 1 (4.55 %) recurred. In the low SDF-1α expression group, 4 (6.15 %) recurred. In the high SDF-1α expression group, 18 (18.00 %) recurred. These data illustrated a lower trend of recurrence for the negative expression group than for the low expression and high expression groups (*χ*^2^ = 6.891, *P* = 0.032; Fig. 4c).

To evaluate the prognostic factors related to EC patients’ survival, hazard ratios (HRs) were estimated using multivariate Cox regression analysis as shown in Table 2. After adjusting for age at diagnosis, menopausal status, tumor histopathologic grade, histopathologic subtype, International Federation of Gynecology and Obstetrics (FIGO) stage, and lymph node metastasis, we found that high expression of SDF-1α (HR, 4.13; 95 % confidence interval (CI), 1.58–11.07; *P* = 0.005) and deep myometrial invasion (HR, 2.77; 95 % CI, 1.08–7.09; *P* = 0.034) were independent factors predicting cancer-specific survival in EC patients (Table 2). Altogether, our findings indicated that SDF-1α expression might be a useful marker for predicting the survival of patients with EC.

**Table 2:** Multivariate analysis of factors associated with survival of EC patients

Variables		HR	95 % CI	Wald value	*P*
Age (years)	<60	1.00	0.55–3.43	0.45	0.501
	≥60	1.37			
Menopausal status	Yes	1.00	0.18–1.90	0.83	0.363
	No	0.58			
Histologic subtype	Endometrioid	1.00	0.67–7.82	1.74	0.187
	Nonendometrioid	2.29			
Histopathologic grade	G1	1.00	0.55–2.97	0.31	0.578
	G2–G3	1.27			
Myometrial invasion	<1/2	1.00	1.08–7.09	4.50	0.034^*^
	≥1/2	2.77			
FIGO stage	I–II	1.00	0.43–3.99	0.22	0.639
	III–IV	1.31			
Lymph node metastasis	Yes	1.00	0.22–2.27	0.335	0.563
	No	0.71			
SDF-1α expression	Low expression	1.00	1.58–11.07	7.918	0.005^*^
	High expression	4.13			

*P < 0.05

### Expression of CXCR4 in EC

Furthermore, we investigated CXCR4 expression in xenotransplanted tumors, as well as in NE, HE, AHE, and EC tissue microarrays. CXCR4 immunostaining was more intense in the endometrial epithelium than in the stroma, and its staining was located in the membrane. CXCR4 expression was stronger in the xenograft tumor containing CAFs compared to tumors without fibroblasts or containing NFs (Fig. 2f) and increased gradually in endometrial tissues as they progressed from NE to HE to AHE and finally to EC tissues (*χ*^2^ = 139.485, *P* = 0.000; Fig. 4d). Additionally, CXCR4 expression was higher in EC tissues than in NE tissues (*P* = 0.000), in HE tissues (*P* = 0.000) and in AHE tissues (*P* = 0.000), whereas there were no differences between AHE and HE tissues (*P* = 0.111), between AHE and NE tissues (*P* = 0.172), or between HE and NE tissues (*P* = 0.443).

We also determined that CXCR4 expression in EC tissues was not correlated with any prognostic factors. Finally, we found that there was no association between CXCR4 expression in EC tissues and overall survival.

## Discussion

Myofibroblast-rich cell populations, originally introduced as CAFs, represent one of the most abundant stromal cell types in various type of cancer, including EC. Different cellular origins and tumor-derived factors affect the phenotype of CAFs and contribute to their appearance as heterogeneous cell populations with distinct subtypes [13]. Some studies have reported a role of promoter in tumor growth and progression [24–28]; however, recent data obtained from in vitro cocultures and in vivo xenograft models have shown a tumor-inhibitory role of CAFs [29–32]. It remains unknown whether CAFs in EC exhibit pro-malignant properties or anti-malignant characteristics. To clarify this role, CAF cell population and NF cell population were established from human EC tissues and normal endometrial tissues. The present study revealed that CAFs in EC were different from the NFs in several important functional respects. (1) CAFs were more competent than the NFs in enhancing tumor growth, migration, and invasion by comingling with EC cells in in vivo and in vitro experiments. (2) CAFs produced increased level of SDF-1α and (3) CAFs promoted EC cells progression via the SDF-1/CXCR4 axis in a paracrine-/autocrine-dependent manners. (4) SDF-1α expression was upregulated and was associated with EC progression and poor prognosis, while the increased expression of CXCR4 in EC tissues was not correlated with any prognostic factors.

As we know, few studies have used CAFs from human EC samples due to the relative paucity of available fresh tumor specimens and the limited life span of primary cells [33]. CAFs or NFs grow slowly and eventually senesce after 10 to 15 passages; therefore, all of the primary cells in our study were obtained at earlier than 10 passages, to maintain the closest phenotype to the primary tissues. Our data showed that CAFs, distinctly differently from NFs, exhibited a pro-tumorigenic effect by coculture with EC cells. Similar to Olumi’s finding [5], human prostatic CAFs grown with initiated human prostatic epithelial cells dramatically stimulated growth and altered histology of the epithelial population. This effect was not detected when NFs grown with initiated human prostatic epithelial cells under identical conditions. Furthermore, studies have demonstrated that stromal cells isolated from proliferative NE are capable of suppressing the growth of the EC cell line (Ishikawa), even in response to estrogen [34, 35]. Such effects were specific to the fibroblasts derived from normal endometrium because fibroblasts from normal foreskin failed to exhibit similar effects [34]. Thus, different fibroblasts subtypes display different phenotypes. However, it is less clear whether fibroblast subpopulations stimulate distinct aspects during different stages of malignancy.

CAFs can promote tumor growth, angiogenesis, and metastasis through communication with cancer cells, such as by secreting various important factors in paracrine- or autocrine-dependent manners. Among these factors, SDF-1 has sparked substantial interest because of its pro-tumorigenic role. SDF-1 has two major isoforms, α and β. Both are derived from a single gene, due to alternative splicing. SDF-1α is the predominant isoform, and it is secreted by stromal cells and is found nearly in all the organs [36]. The chemokine SDF-1 is an important α-chemokine that binds to its cognate receptor CXCR4 and regulates the trafficking of normal and malignant cells [37]. Thus, we chose the SDF-1α as a target in this research. We demonstrated that SDF-1α released by CAFs induced cell proliferation, mobility, and invasiveness in EC cells. At the same time, AMD3100, a synthetic antagonist known to block CXCR4 function [38], could significantly suppress these effects, consistent with its action in other malignancies [20, 39].

Schmidt et al. have observed that SDF-1α induced the development of EC cells in vitro [40]. However, to date, activation of the intracellular signal transduction induced by SDF-1α in EC has been seldom reported. Tsukamoto and colleagues demonstrated that SDF-1α mediated the activation of the PI3K/Akt pathway, but not the MAPK/Erk pathway, in EC cells after treatment with supernatants from uterine smooth muscle cells [41]. Zhao et al. reported that stimulation with exogenous SDF-1α could induce EC cell growth through activating PI3K/Akt and MAPK/Erk pathways in a dose-dependent manner [42]. Previous studies have documented a direct involvement of PI3K/Akt and Erk signaling pathways in cardioprotection and chemotherapy resistance mediated by the SDF-1/CXCR4 axis [43, 44]. Zhuo’s group has stated that SDF-1 increased phosphorylation of Akt and Erk1/2 in mouse lymphatic endothelial cells, when CXCR4 was blocked by its neutralizing antibody or knocked down by effective small interfering RNA (siRNA) eliminated the effect of SDF-1 on activated of Akt and Erk1/2 [45]. Similarly, these two pathways were both activated by secretions from CAFs in our study. In addition, AMD3100 could inhibit SDF-1α-induced Akt and Erk activation. Our findings elucidated that SDF-1α-induced intracellular signaling activation was a downstream effect of CXCR4. These observations were also compatible with changes in the biologic responses, such as cell growth, migration, and invasion, to CAF-conditioned media treatment or to AMD3100 pretreatment of EC cells. Therefore, one potential mechanism of CAFs promoting EC development is that SDF-1α is engaged in tumorigenesis in a paracrine-dependent manner.

Metastasis is an organized sequence of events beginning with the detachment of neoplastic cells from a primary tumor and their entry into the circulation, dissemination, and arrest at select organs [46]. A degradation of basement membrane is the first step toward invasion and metastasis. Type IV collagen is the main component of basement membrane, and destruction of this structural protein is favored by two MMPs, namely gelatinase A (MMP-2) and gelatinase B (MMP-9) [47]. These MMPs are known to be closely associated with the malignant potential of tumor cells. CAFs affect cancer cell invasion by both cell-cell contact and pro-invasive factor secretion. CAFs are also one of the most significant contributors to MMP production [48]. Koontongkaew et al. reported that direct contact between tumor and fibroblast cells was required to activate MMP-2 and MMP-9 secretion in both tumor cells and fibroblasts. Moreover, it was demonstrated that fibroblasts seemed to be responsible for the increased MMP-2 in the coculture. In addition, fibroblast- or tumor cell-conditioned media upregulated the secretion of MMP-2 and MMP-9 in HNSCC cells. These findings indicated that the SDF-1/CXCR4 signaling pathway might cause an increase in cellular motility, as well as MMP-2 and MMP-9 activation, in autocrine- and paracrine-dependent manners [49]. However, we simply found that the SDF-1/CXCR4 axis played a role in the invasion and metastasis of EC in an autocrine-dependent mode.

We investigated and clarified the clinical significance of SDF-1α expression in EC. The data showed that SDF-1α expression was upregulated and was associated with tumor progression. In addition, patients with high SDF-1α expression showed significantly poorer oncologic outcomes than patients with negative and low expression, suggesting that SDF-1α is an independent prognostic factor in EC. In contrast, no significant associations were documented between the expression of CXCR4 and the clinicopathologic characteristics of EC. Similarly, this parameter was not proved to play role in the negative prognosis with this malignancy. The number of published reports on the prognostic value of the SDF-1/CXCR4 axis in EC patients is small [50]. Furthermore, the results of these studies have been inconclusive and sometimes contradictory. Mizokami et al. found reverse correlation between histological grade and the stromal expression of SDF-1 and CXCR4 in 41 EC cases [51]. Another study showed that positive SDF-1 expression was associated with longer overall survival and longer recurrence-free survival in ER-negative patients [52]. However, in a recent study in 92 patients with EC, the authors revealed that higher expression of SDF-1/CXCR4 axis components was associated with worse prognosis [17]. These evidences suggest that the role of the SDF-1/CXCR4 axis in the progression of EC remains undefined. It might be these studies adopted different sets of prognostic factors and various classification systems for protein expression.

## Conclusions

Taken together, our results suggest that CAFs derived from EC tissues promoted EC progression via the SDF-1/CXCR4 axis. Furthermore, SDF-1α expression emerged a novel independent poor prognostic factor for predicting survival in patients with EC. Thus, suppression of the SDF-1/CXCR4 axis in a primary tumor environment might be a target for therapy in EC patients.

## Methods

### Ethics statement

This study was approved by the Ethics Committee of Tianjin Medical University General Hospital. Written informed consent was obtained from all participants.

### Isolation and culture of primary fibroblast cells/cell lines

To isolate stromal fibroblasts, primary cancer tissues were obtained from 12 EC patients at Tianjin Medical University General Hospital (Tianjin, China). These patients had undergone hysterectomy but had not been treated with preoperative chemotherapy or radiotherapy. Ten NE tissues were obtained from women undergoing surgery to remove their uteruses because of leiomyomas. The tissues were divided into two parts for histopathological diagnosis and isolation of stromal fibroblasts. The EC tissues used for isolation of stromal fibroblasts were diagnosed as endometrioid adenocarcinomas. The fresh tissues were transported to the laboratory in media consisting of DMEM/F12 (Gibco, USA) supplemented with 10 % fetal bovine serum (FBS) (Gibco, USA) and 1 % penicillin/streptomycin (Life Technologies, USA). The tissues were minced to the size of 1 mm^3^ and then were digested with collagenase I (2 mg/ml; Sigma, USA), collagenase II (2 mg/ml; Sigma, USA), and hyaluronidase (50 μg/ml; Sigma, USA) in DMEM/F12, using a rotator for approximately 45 min at 37 °C. Post-digestion, the tissues were washed and cultured in DMEM/F12 media supplemented with 10 % FBS and 1 % penicillin/streptomycin at 37 °C. The cultures were maintained by media changes every 72 h, and subculture was performed after the cultures reached confluency. After two to three passages, a unique homogeneity of stromal fibroblasts was formed. All the stromal fibroblasts used in the experiments were at less than 10 passages, to maintain the closest phenotype to the primary tissues. The human EC cell lines ECC-1 (CRL-2923) and HEC-1B (HTB 112) were purchased from American Type Culture Collection (Bethesda, MD, USA) and were cultured in media according to manufacturers’ protocols.

### Identification of fibroblast cells

Primary cultured fibroblasts from human EC and human NE were also confirmed by immunocytochemistry using rabbit anti-FSP-1 polyclonal antibody (1:300; Abcam, UK), rabbit anti-FAP polyclonal antibody (1:500; Abcam, UK), mouse anti-α-SMA monoclonal antibody (1:300; Sigma, USA), rabbit anti-CK monoclonal antibody (1:500; Abcam, UK), rabbit anti-vimentin polyclonal antibody (1:400; Abcam, UK), and rabbit anti-CD31 polyclonal antibody (1:200; Abcam, United Kingdom). The cell seed sections were then treated with 0.3 % hydrogen peroxide (H_2_O_2_) in water for 10 min to quench any endogenous peroxidase activity within the tissue, and the nonspecific binding sites were blocked with 0.5 % bovine serum albumin for 10 min at room temperature. Next, the sections were incubated for 45 min in the presence of the primary antibody, and then the slides were washed in phosphate-buffered saline (PBS) containing 0.1 % Tween 20 (PBS/Tween) for 15 min, with the solution changed three times before the application of the secondary biotinylated antibody. The slides were incubated with the secondary antibody for 30 min at room temperature before being washed for 15 min in PBS/Tween, which was changed three times. The sections were then incubated for 15 min with an avidin-biotinylated horseradish peroxidase complex, and the reaction was visualized using 0.02 % 3,3′-diaminobenzidine tetrahydrochloride as a chromogen in a Tris-HCl buffer, at pH 7.6, containing 0.03 % H_2_O_2_. Hematoxylin was used to counterstain the nuclei.

### Preparation of conditioned media

CAFs/NFs were seeded and cultured in complete media for 24 h, before being cultured in media with serum-free DMEM/F12 for the subsequent 72 h. Conditioned medium was collected and centrifuged at 1000×*g* at 4 °C for 15 min, and the supernatant was concentrated with Centricon YM3 filters (Milipore). The protein in the concentrated media was quantified using Bradford assay (Biorad, CA, USA).

### ELISA assay

CAFs or NFs were seeded on six-well plates at a density of 1.5 × 10^5^ cells in DMEM/F12 with 10 % FBS. After 12 h of incubation, the media was changed to 500 μl of serum-free DMEM/F12, followed by incubation for 24, 48, and 72 h. The conditioned media was detected using ELISA kits, according to the manufcturers’ protocol.

### Coculture and cell proliferation assay

A total of 1.5 × 10^3^ NFs or CAFs were mixed with 4.5 × 10^3^ HEC-1B cells or ECC-1 cells and were seeded in complete media in 96-well plates. At 24 h post-seeding, the cells were treated with serum-free media, AMD3100 (Sigma, USA) was added to cells at varying concentrations (0, 200, 500 ng/ml). Serum-free DMEM/F12 was added to control wells. Cell growth was analyzed at 24, 48, 72, and 96 h with the MTT reagent (Promega) added 4 h before performing spectrophotometric reading, according to the manufacturer’s directions.

### Cell migration and invasion assays

Cell invasive and migrative abilities were determined using transwell chambers coated with or without extracellular matrix gel (BD Biosciences, USA). A total of 1 × 10^5^ cells/well were seeded on the upper inserts with 8-μm pores (BD Biosciences, USA) and were cultured with serum-free media. In the lower chamber, 1 × 10^5^ NFs or CAFs in 500 μl of serum-free media were planted. In the control group, there were only 500 μl of serum-free media without fibroblasts in the lower chamber. Furthermore, various concentrations of AMD3100 were added to the lower wells. After 24 h of incubation, the cells on the upper surface of the filters were removed; the filters were fixed with 4 % paraformaldehyde for 15 min and were stained with crystal violet stain for 30 min (Sigma, USA). The invasive and migrative activity was quantified by counting the number of transpassed cells in five random regions (magnification, ×200) by two independent observers who were blinded to the data. Migration and invasion assays were run in triplicate, and the data were expressed as the average number of cells per random area.

### Nude mice xenograft assays

All of the experimental animal procedures were approved by the Animal Care and Use Committee of Tianjin Medical University. Four-week-old female athymic nude mice were maintained in individually ventilated caging systems in groups of five. Established stable cells (6 × 10^6^ NFs or CAFs mixed with 2 × 10^6^ HEC-1B cells) were injected subcutaneously into the right flank of each mouse. Tumor growth was monitored at 5-day intervals by measuring the length and width of the tumor with calipers and calculating the tumor volume based on the following formula: volume = 0.5 LW^2^. The mice were sacrificed, and the tumors were harvested and measured.

### Western blot analysis

HEC-1B cells were seeded at 1 × 10^4^ cells/well in six-well plates in complete media. At 24 h post-seeding, the cells were treated with NF-conditioned media, CAF-conditioned media, and/or AMD3100 (200 or 500 ng/ml) for 1 h. Cell lysates or immunoprecipitates from cell lysates were subjected to SDS-PAGE and were transferred to polyvinylidene fluoride membranes. The membranes were incubated with the following primary antibodies: rabbit anti-human Akt, phospho-Akt, Erk, phospho-Erk, and GAPDH (Cell Signaling Technology, USA), followed by horseradish peroxidase-conjugated secondary antibody. The immunoreactive polypeptides were visualized using a chemiluminescent substrate (GE Life sciences).

### Zymography

Conditioned media, standardized for cell numbers, were mixed with equal volumes of nonreducing sample buffer and were resolved on 10 % Novex Zymogram gels containing 0.1 % gelatin (Sigma, USA). Renaturation and detection were performed according to the manufacturer’s instructions. Clear bands corresponding to gelatinolytic activity were measured by densitometry.

### Patients and samples

All of the cases examined in this study were obtained from surgically removed tissues of inpatients in Tianjin Medical University General Hospital (Tianjin, China) from 2000 to 2012. The patients were confirmed by histopathology, and none of them underwent radiotherapy or chemotherapy prior to surgery. A total of 348 patients were approached. Controls were selected from women who presented for routine examination in the Department of Regular Physical Examination Center or for uterine prolapse, cystocele, or urethrocele. Among these cases, 202 patients had EC, 26 patients had HE, 33 patients had AHE, and 87 patients had NE. The histological type and grade of the primary tumors were determined by two independent pathologists based on a modified WHO classification system, whereas EC staging was performed based on a modified 2009 FIGO staging system.

### Tissue microarray

All of the tissues for tissue microarray were obtained from formalin-fixed, paraffin-embedded tissue blocks. All of the cases were histopathologically re-evaluated and their tumor content verified on hematoxylin-eosin-stained slides. Representative areas of tumor and normal tissue were selected to be cored. The tissue microarray was designed and constructed using MTA Booster and TMA Designer. The sample spots were designed to be 1.5 mm in diameter and to range in length from 2.0 to 5.0 mm, depending on the depth of tissue in the donor block. A total of 48 such cylindrical cores were precisely arrayed in one recipient block.

### Immunohistochemistry

The streptavidin-peroxidase-biotin immunohistochemical staining method was used to study the expression of SDF-1α and CXCR4 in tissue microarray samples. Briefly, paraffin-embedded specimens were cut into 4-μm sections and were baked at 60 °C for 1 h. Endogenous peroxidase activity was quenched by incubation in 3 % hydrogen peroxide/methanol buffer for 30 min. The sections were incubated in rabbit anti-SDF-1α polyclonal antibody (1:200; Abcam, UK) or rabbit anti-CXCR4 polyclonal antibody (1:400; Abcam, UK) overnight at 4 °C in humidified chambers. The following day, the sections were washed three times in PBS and were incubated in a peroxidase-conjugated goat anti-rabbit IgG antibody, which came from the streptavidin-peroxidase-biotin reagent kit, for 30 min at 37 °C. After being washed in PBS, the tissue sections were stained with diaminobenzidine, counterstained with hematoxylin, and then examined under a light microscope. As negative controls, tissue sections were processed as described above, except that they were incubated overnight at 4 °C in blocking solution with PBS.

### Scoring of immunohistochemical staining

SDF-1α and CXCR4 staining was scored based on the percentage and the intensity of positively stained cells. The five score categories for positive staining percentage were as follows: 0, no positive cells; 1, 25 % or fewer positive cells; 2, 26 to 50 % positive cells; 3, 51 to 75 % positive cells; and 4, 76 % or more positive cells. The four score categories for staining intensity were as follows: 0, no intensity; 1, weak intensity; 2, moderate intensity; and 3, strong intensity. SDF-1α and CXCR4 expression was determined by adding the positive staining percentage score to the intensity score: 0, negative expression; ≤4, low expression; and >4, high expression. The staining was determined independently by two pathologists, who were blinded to the patients’ clinicopathologic information.

### Statistical analysis

Group comparisons of categorical variables were performed using the *χ*^2^ test. Comparisons of average means were performed with Student’s two-tailed *t* test or one-way ANOVA. Cancer-specific survival was defined from the date of surgery to the date of death from EC. Survival curves were plotted using the Kaplan-Meier method and were analyzed using the log-rank test. A Cox proportional hazards model was created to identify prognostic factors for survival. *P* < 0.05 was considered to be statistically significant. All of the statistical analyses were conducted using SPSS statistical software (SPSS Inc., Chicago, IL, USA), version 14.0.

## Abbreviations

AHE: atypical hyperplastic endometrium
CAFs: cancer-associated fibroblasts
CK: cytokeratin
CSF-1: colony stimulating factor-1
CXCR4: chemokine receptor 4
EC: endometrial cancer
FAP: fibroblast-activating protein
FBS: fetal bovine serum
FSP-1: fibroblast-specific protein-1
HE: hyperplastic endometrium
IL-1: interleukin-1
MCP-1: macrophage chemoattractant protein-1
MIF: migration inhibitory factor
MMP: matrix metalloproteinases
NE: normal endometrium
NFs: normal fibroblasts
PBS: phosphate-buffered saline
SDF-1α: stromal cell-derived factor-1alpha
α-SMA: alpha-smooth muscle actin
